# Scale-Free Navigational Planning by Neuronal Traveling Waves

**DOI:** 10.1371/journal.pone.0127269

**Published:** 2015-07-09

**Authors:** Azadeh Khajeh-Alijani, Robert Urbanczik, Walter Senn

**Affiliations:** 1 Department of Physiology, University of Bern, Bern, Switzerland; SUNY Downstate MC, UNITED STATES

## Abstract

Spatial navigation and planning is assumed to involve a cognitive map for evaluating trajectories towards a goal. How such a map is realized in neuronal terms, however, remains elusive. Here we describe a simple and noise-robust neuronal implementation of a path finding algorithm in complex environments. We consider a neuronal map of the environment that supports a traveling wave spreading out from the goal location opposite to direction of the physical movement. At each position of the map, the smallest firing phase between adjacent neurons indicate the shortest direction towards the goal. In contrast to diffusion or single-wave-fronts, local phase differences build up in time at arbitrary distances from the goal, providing a minimal and robust directional information throughout the map. The time needed to reach the steady state represents an estimate of an agent’s waiting time before it heads off to the goal. Given typical waiting times we estimate the minimal number of neurons involved in the cognitive map. In the context of the planning model, forward and backward spread of neuronal activity, oscillatory waves, and phase precession get a functional interpretation, allowing for speculations about the biological counterpart.

## Introduction

Planning is a hallmark of higher cognitive functions. It has been particularly well studied as navigational planning that involves hippocampal-prefrontal cortex structures, and the neuronal processing involved in this case was suggested to be paradigmatic for planning in general [1]. Medial temporal lobe has been proposed to form a cognitive map [2, 3]. Yet, how navigational planning is possible in a noisy neuronal substrate remains an open question.

From a computational perspective, navigational planning amounts to finding the shortest route between two points. This can be formalized in terms of a path search problem in a graph specified by nodes and connections. There is a set of optimal algorithms solving this problem that go back to the classical breath-first search algorithm by Dijkstra [4]. In its backward version, this algorithm determines the distances from a target node backwards to successive neighbors throughout the graph until the start node is reached, and from there works stepwise forward to the target node again [5]. A bidirectional version of breath-first graph search algorithm is also implemented by simultaneously triggered waves of activity at both the target and the start node that propagates through multiple networks by diffusive coupling [6].

**Fig 1:**
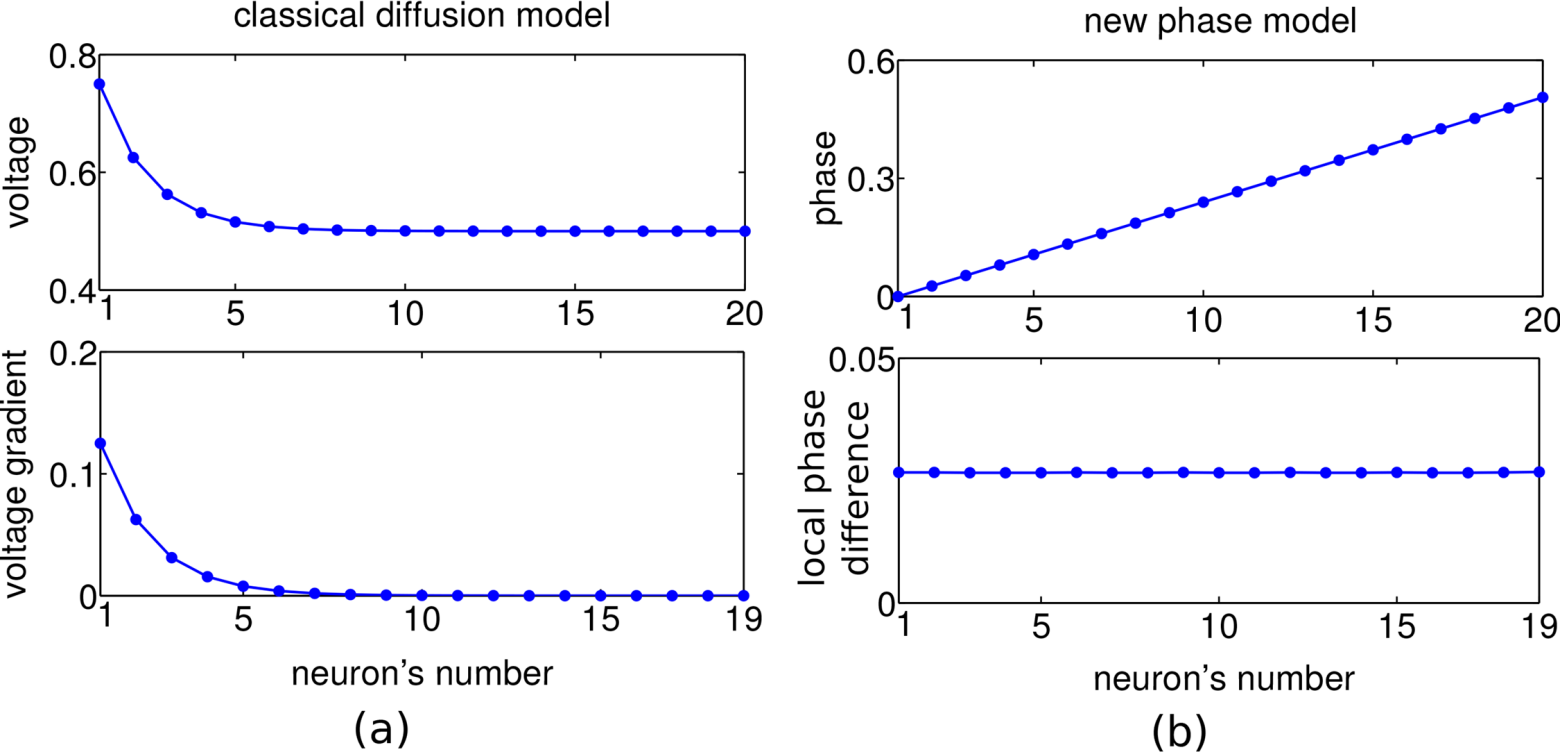
A comparison of the classical diffusion model and our scale-free traveling wave model in 1 dimension. For illustration, a linear environment with 20 discrete neurons and goal at position 1 is considered. In the diffusion model (a), activity (membrane voltage) is spread from the goal across the environment (top panel) with exponentially decaying gradient, and hence quickly fading directional information (bottom). In the traveling wave model (b), activation of the goal synaptically spreads through the environment such that the firing phase of adjacent neurons increases linearly with distance from the goal (top), resulting in a fixed and positive local phase difference and hence in directional information that does not decay in space and is not restricted to a specific scale (‘scale-free’), bottom.

In some form such a 2-step backspread–forwardtrack procedure is present in all of today’s graph search algorithms, and it is difficult to imagine solutions of the planning problem which do not involve this core idea. Accordingly, various neuronal planning models have considered the backpropagation of activity from the goal across a topological map of the environment towards the start position [7–11]. However, these models suffer from an exponential decay of activity with distance from the goal. In technical solutions, evaluating small signals just requires high numerical range and precision. But in biological systems the large neuronal fluctuations prevent a reliable implementation across multiple spatial scales (Fig 1a). A recent proposal considers the spread of a single front of action potentials across a topographic map [12]. The direction from which the front reaches the start position first, indicates the shortest path. Yet, if independent noise is added by each neuronal processing step, information is again lost quickly. Here we suggest a phase-coding scheme that allows an agent to plan within a single network across many spatial scales, without requiring a hierarchical coding [13].

## Results

We first outline the basic idea in the 1-dimensional case. The various models that assume a spread of directional information from the goal to putative starting points via diffusion of activity [7–11] inherently set a spatial scale for the planning that is limited by the length constant of the exponential activity decay. In fact, in the presence of a certain noise level and an upper bound of the activity at the goal position, the directional information will vanish after a few multiple of this length constant (Fig 1a).

To overcome the problem of exponential information decay (Fig 1a), we considered an encoding of the directional information in the local phase differences of a periodic traveling wave spreading across the map. All map neurons receive a synaptic drive that fires them periodically, with the goal-representing neuron being driven stronger and thus firing initially with a slightly higher frequency. Due to the recurrent nearest neighbor connectivity the surrounding neurons one after the other will adapt to this faster frequency and, eventually, the map neurons all fire with the same fast period, but with phase shifts that increase with distance from the goal. Hence, a local comparison of the phases allows for detecting the shortest path to the goal. The direction is determined by the neighboring neuron that fires earliest within a cycle, leading to a movement along decreasing phases (Fig 1b, top). Even at large goal distances, the same non-zero local phase difference between neighboring neurons is attained, although more time is needed for converging to the steady state the further away a neuron is from the goal (for a mathematical treatment see S1 Text).

### Encoding goal, positions and obstacles

**Fig 2:**
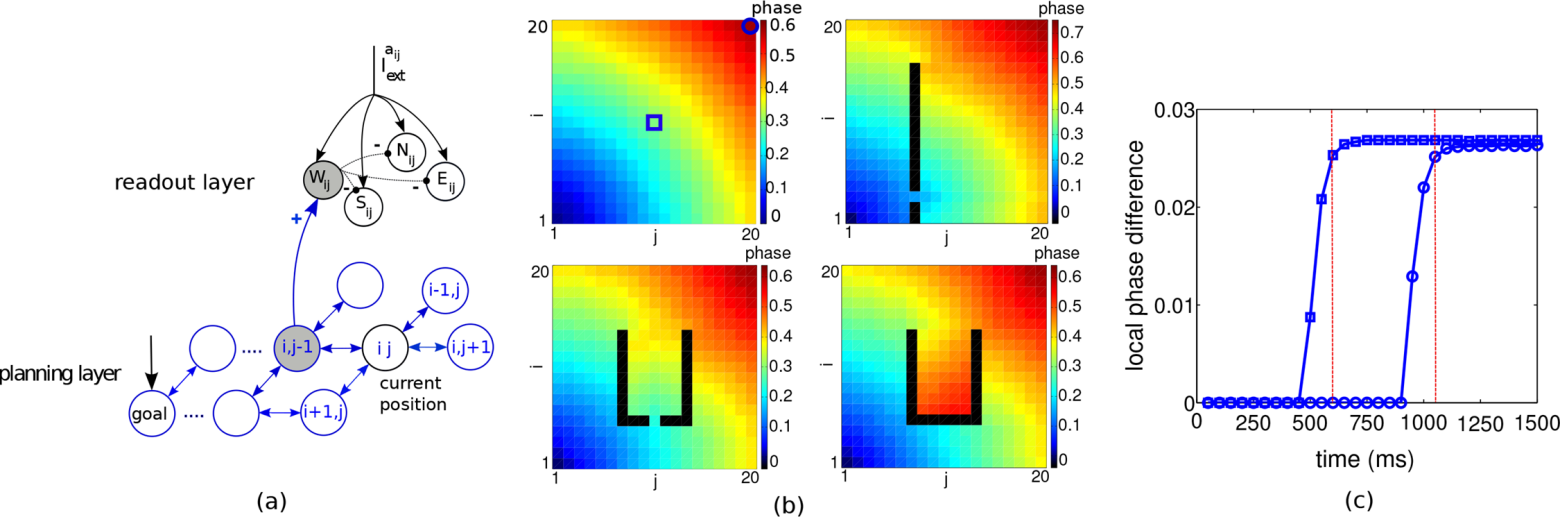
Network architecture, traveling waves and planning time. (a) Planning and readout network. For each neuron in the planning layer, 4 actions can be assigned. Actions neurons associated to planning neuron (*i*, *j*) are *W*_*ij*_, *E*_*ij*_, *N*_*ij*_ and *S*_*ij*_ which receive synaptic input respectively from the left, right, north, and south neighbor of the neuron (*i*, *j*) and evoke a motion in the same directions (just one synaptic input is shown). Action neurons corresponding to the current place of the agent (here again (*i*, *j*)) are driven by an additional input ($I_{ext}^{a_{ij}}$). The first of the 4 action neurons that is fired by the passing traveling wave inhibits the other 3 action neurons. (b) Synaptically propagating waves of activity from the goal neuron at (1,1) across a planning layer of 20 × 20 neurons, for four different obstacle configurations. Colors code for firing phases at steady state relative to the goal neuron. (c) Time courses of the local phase difference for two sample neurons at positions (10,10) and (20,20), indicated by □ and ○ in the top left panel of b, with their local west-positioned neighbors. The time to reach the maximal local phase difference represents the planning time for these two start positions towards the goal (here 600 and 1050 ms, vertical lines), and subsequently the full path towards the goal can be read out.

We next consider a 2-dimensional well-explored environment with possible obstacles. The planning layer is composed of spiking neurons each coding for a position in the environment. Excitatory synaptic connections between neurons exist if the places the neurons represent are adjacent to each other (Fig 2a). The readout layer associates 4 cardinal motion directions to each place in the planning layer. It reads out the local phase differences in the planning layer and translates the readings into a sequence of actions towards the goal.

For the planning layer, we consider an array of *N* × *N* synaptically coupled, single compartment Hodgkin-Huxley type neurons. The dynamics of the membrane potential *V*_*ij*_ of the planning neuron encoding position (*i*, *j*) is given by

$$\begin{array}{c} C\frac{dV_{ij}}{dt}=-I_{L}^{ij}-I_{ion}^{ij}+\epsilon I_{syn}^{ij}\left(t\right)+I_{ext}^{ij}\left(t\right)\;,\;\;\;i,j=1,2,\cdots N\;. \end{array}$$ (1)

Here, $I_{L}^{ij}$ is a leak current, $I_{ion}^{ij}$ defines the various intrinsic membrane currents responsible for the action potential generation, $I_{syn}^{ij}$ is the synaptic current from the 4 neighboring neurons defining whether or not the place (*i*, *j*) can be reached from that neighbor (for details see Materials and Methods).

The external current $I_{ext}^{ij}(t)$ is produced by a population of *N*_E_ external neurons that stochastically fire with a certain Poisson rate *ν* and have an excitatory connection strength *J*. By choosing these parameters, we can independently tune the mean *μ*_*ext*_ and the standard deviation *σ*_*ext*_ of the external current (see also Materials and Methods). This allows us to control the level of noise in the simulations. The goal neuron is driven by a slightly stronger external current than the other planning neurons ($\mu_{ext}^{goal}>\mu_{ext}$). In the absence of a recurrent synaptic coupling (*ϵ* = 0), each neuron *ij* fires periodically with some jitter in the firing times. When the goal neuron has larger intrinsic oscillation frequency ($\mu_{ext}^{goal}>\mu_{ext}$), weak coupling (*ϵ* > 0) shifts the firing phases of its neighbors and this progressively influences the firing phases of other neurons. This frequency difference propagates through the network and leads to a periodic traveling wave [14–16]. For a neuron representing a transient obstacle, the external drive inhibits it such that it does not fire, even when receiving the weak lateral input.

### Firing phase encodes distance to the goal

When turning on the external input to the goal and the other neurons, the planning network organizes itself into a periodic traveling wave spreading from the goal neuron through the network. After a transient period, the firing phases become ordered according to their spatial distance to the goal, i.e. spatially closer neurons to the goal fire earlier than those far away (Fig 2b). This situation repeats itself after a fixed time, the common period of the population. This periodic traveling wave is a stable phase-locked state in which all the neurons periodically fire with a common period but with a phase difference in the firing times [17, 18].

Fig 2b shows examples of stationary phases of a periodic traveling wave spreading from the goal neuron at position (1,1) across the planning layer with and without obstacles. The intrinsic frequency of the goal neuron is 18 Hz and that of the other neurons 17 Hz. The development of the local phase differences for two distal neurons, after injecting the small additional current into the goal neuron, shows a delayed propagation of the directional information with a swift increase and convergence to the steady state after 600 and, respectively, 1050 ms (Fig 2c). The steady state of the traveling wave is said to be reached when the increase of the local phase difference at two successive times is less than 10% of the current value. The time from turning on the external neurons until the steady state at the start position is referred to as planning time. Thereafter, the spatial ordering of the firings is established between start and goal, so that firing phases can be read out across the entire path without requiring additional planning. Remarkably, for readout it is necessary that a non-zero local phase difference is reached independently of the distance from the goal (see Materials and Methods). This feature assures a noise-robust readout of the directional information at any point in the map without information loss with increasing distance from the goal.

### Reading firing phases and local phase differences

Associated to each position, four action neurons representing the cardinal directions (W, E, N, S) receive inputs from the planning layer and an additional input representing the agent current position. In the example of Fig 2a where the agent is assumed to be at position (*i*, *j*), the four action neurons receive the common subthreshold input $I_{ext}^{a_{ij}}$.

When the traveling wave spreading from the goal through the planning layer arrives at the closest neighbor from (*i*, *j*) towards the goal (the left neuron at (*i*, *j* − 1)), the synaptic input from the planning layer to the readout layer fires action neuron *W*_*ij*_ first (Fig 2a). The other three action neurons (*E*_*ij*_, *N*_*ij*_ and *S*_*ij*_) at that position are inhibited by the spiking of the first action neuron. The agent will move towards the new position (*i*, *j* − 1) where the next action can be read out. Note that once the periodic traveling wave reached the steady state at the animal’s position, it did so for all positions towards the goal, and no planning time is required anymore before reading out the action. Hence, at the new place, the next action can be read out from the next period of the wave.

### Background noise is overcome by longer readout times

**Fig 3:**
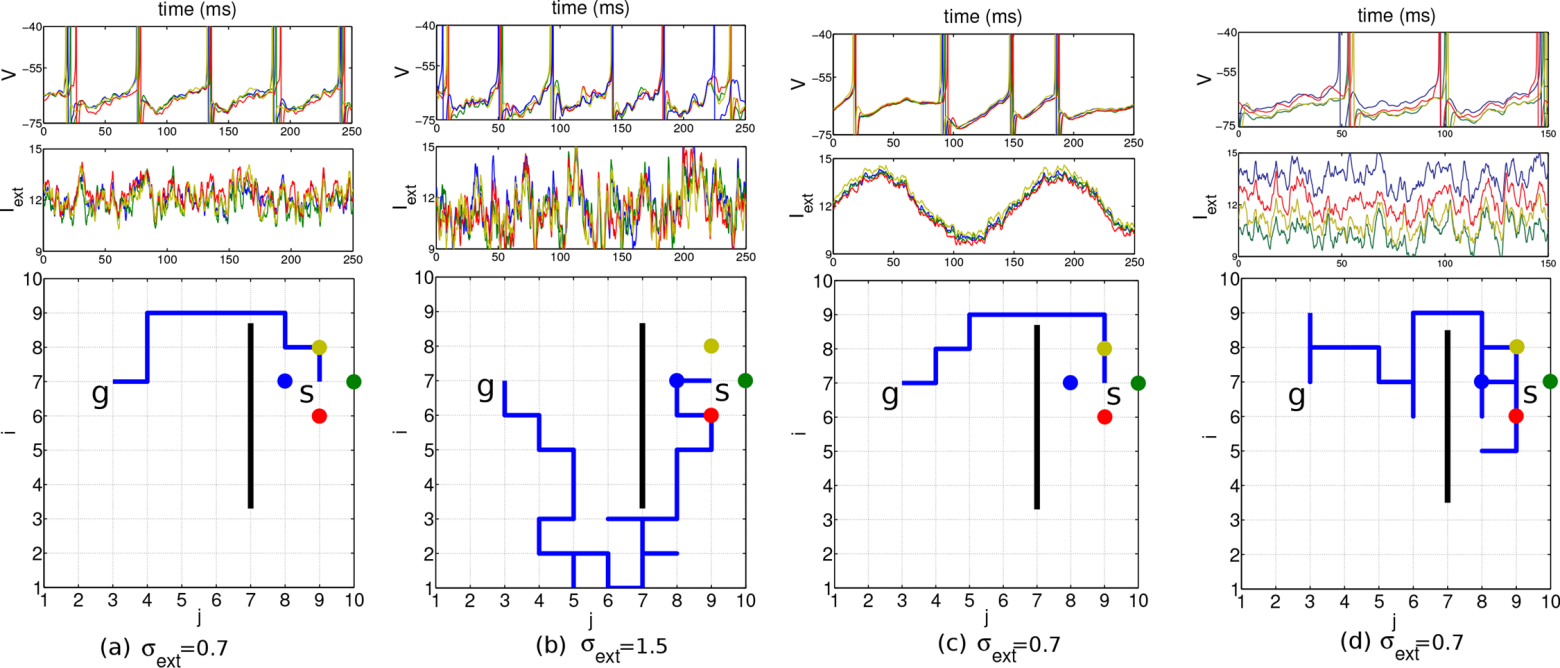
Results in a network of 10 × 10 planning neurons with different noise scenarios. Start position at (7,9), goal position at (7,3) and obstacle indicated by the black bar. Top row: voltage of the four nearest neighbor neurons at the start position (identity color coded). Middle row: external input current these neurons receive. Bottom row: 2D environment with the chosen path (blue line) from the start to the goal. Standard deviations *σ*_*ext*_ of the input currents indicated below. a, b: The mean of the input current generated by the background Poisson spiking neuron was constant and identical for all neurons (*μ*_*ext*_ = 12 mV/ms), except for the goal neuron (marked with *g*, $\mu_{ext}^{goal}=12.5$). c: A common sinusoidal fluctuation in the Poisson firing rate of the background neurons does not disturb the relative timing among neighboring neurons. d: A randomly chosen bias in the mean $\mu_{ext}^{ij}$ of the individual input currents with standard deviation 3 does not prevent the agent from finding a short path to the goal. In all simulations, the planning times were 600 ms, the readout times 250 ms, and the coupling strength was *ϵ* = 0.15.

To check for the noise robustness of our architecture we modulated the noisiness of the external input current. This was achieved by changing the number of external neurons *N*_E_ firing with a constant Poisson rate, and the synaptic strength *J* with which they drive the planning neurons. For instance, a mean *μ*_*ext*_ = 12 mV/ms and standard deviation *σ*_*ext*_ = 0.7 mV/ms of $I_{ext}^{ij}$ is obtained by a total afferent Poisson rate of 16′000 Hz, a synaptic time constant of 2 ms, and a synaptic strength of *J* = 0.375 mV/ms (cf. the voltage trace in Fig 3b, top panel, and Materials and Methods). For such realistic noise the network still displays close to periodic traveling waves and a shortest path can reliably be found (Fig 3a). The external current was driving the goal neurons with a periodicity of roughly 18 Hz and the other planning neurons with 17 Hz. For large values of *σ*_*ext*_, stochastic traveling waves are generated in which the firing times of neurons may become disordered [19] and the readout mechanism fails to find a shortest path, although the goal itself is still found (Fig 3b). In a real brain, the neurons may be subject to a time-dependent common modulation that makes the firing irregular, although it remains correlated (Fig 3c). Furthermore, there may also be a stochastic bias in the external current that drive the individual neurons. This may transiently revert the order of firing among neighboring neurons, but due to the recurrent connectivity, differences in the drive can be corrected and a short path to the goal can still be found (Fig 3d). The bias was produced by randomly varying the input firing rates such that the input currents $I_{ext}^{ij}$ had a temporal mean $\mu_{ext}^{ij}$ that itself was varied.

**Fig 4:**
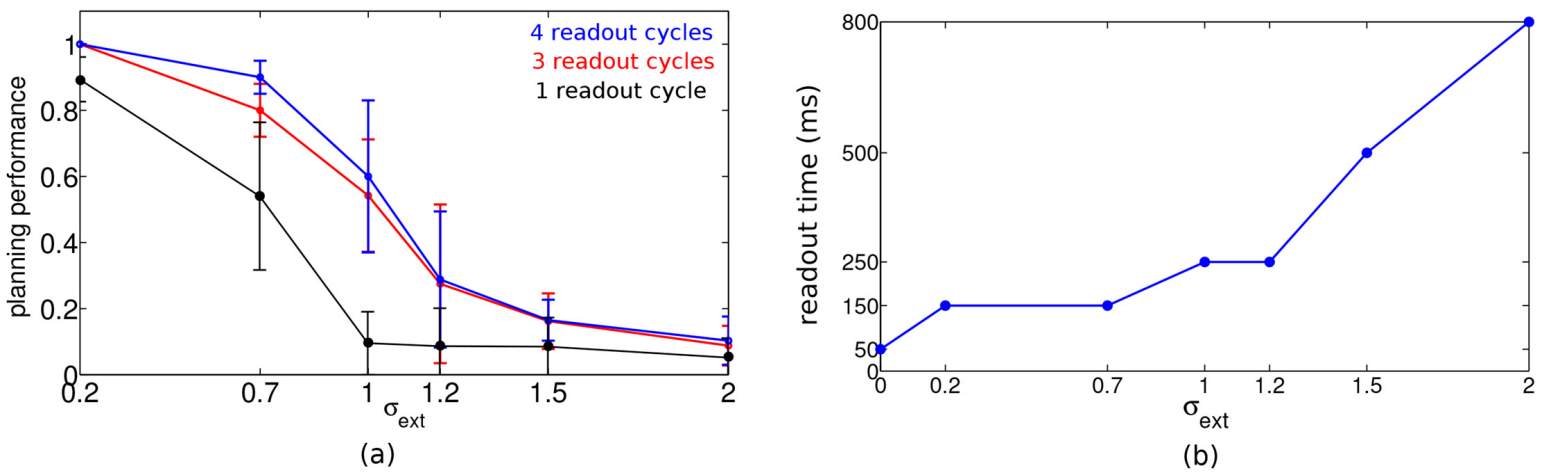
Effect of noise on planning performance and readout time in the network used in Fig 3. (a) Planning performance, shown for 3 different readout times of 60, 180 and 240 ms (corresponding to 1, 3 and 4 readout cycles, bottom to top), declines with increasing noise (average across 10 chosen paths, error bars represent standard deviations of mean). (b) Readout time used at each position such that a shortest 10-step path is found, evaluated for the different noise levels. Parameters, network- and task configuration as used in Fig 3.

To quantify the degradation with noise we introduced a measure of the planning performance (PP) by calculating the ratio between the shortest path connecting start and goal, and the average path lengths that have been chosen, $\text{PP}=\frac{\text{shortest path length}}{\left\langle \text{chosen path lengths}\right\rangle }\;$. The planning performance—here evaluated for start-goal distance of 10 steps—only slowly degrades with the noise level (Fig 4a). The performance in the presence of noise can be improved by extending the readout time for each action selection, i.e. the time for accumulating evidence about the direction to take. The longer this time, the more cycles can be evaluated to check which of the four action neurons associated to the current position typically fires first. With an oscillation frequency of 17 Hz and a readout time of 240 ms, for instance, one obtains 4 readout cycles with an inter-cycle interval of roughly 60 ms, and a considerable improvement in performance as compared to evaluating only a single cycle (Fig 4a). Given a noise level *σ*_*ext*_ we determined the shortest readout time such that the goal is always found on a shortest path in 10 random start-goal configurations of distance 10. The required readout time increases roughly linearly with the noise level (Fig 4b).

### Planning performance increases with frequency

**Fig 5:**
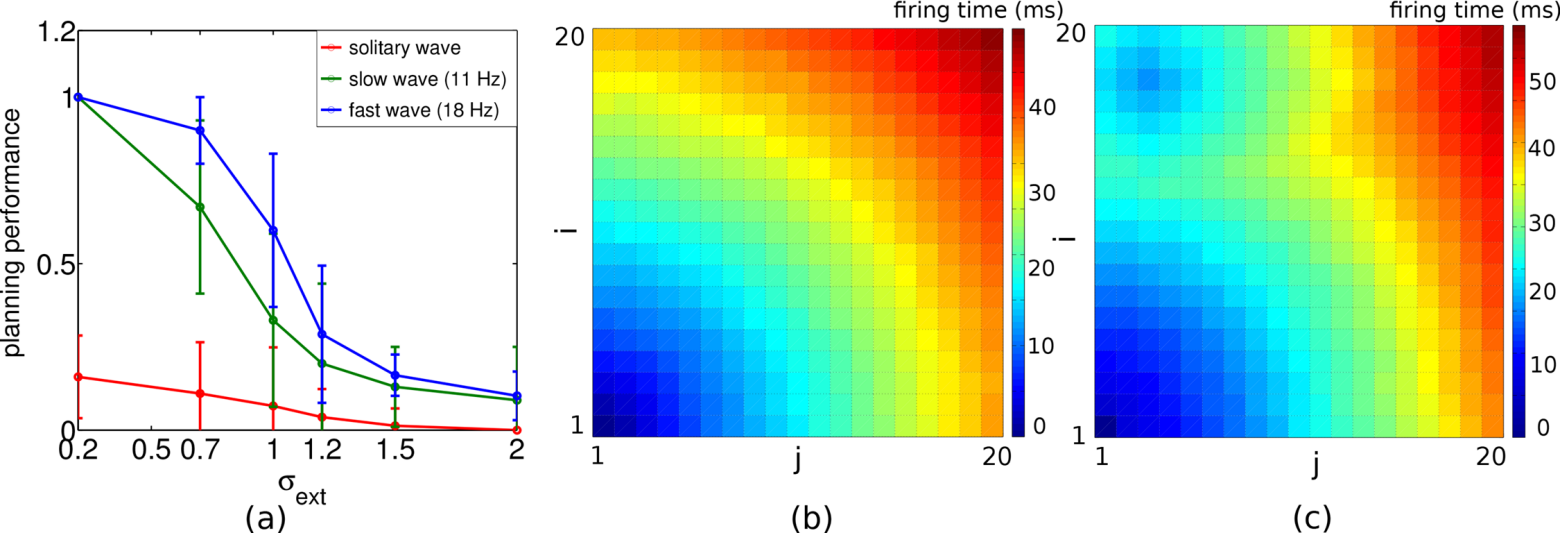
Planning performance decreases with decreasing oscillation frequency and is worst for a solitary wave. (a) Planning performance as a function of the noise level for a 18 Hz (blue) and a 11 Hz (green) intrinsic oscillation frequency with 4 readout cycles to select a single action. For the solitary wave, planning performance was measured after averaging the path lengths across 4 sweeps. Error bars from 10 realizations. (b, c) Color coded spike times relative to the spike time of the goal position (1,1) for (b) the 18 Hz periodic traveling wave after reaching steady state and (c) the solitary wave, both with noise level *σ*_*ext*_ = 0.2. For the solitary wave, the spike times do not faithfully represent distance from goal, and hence the action selection mechanism may yield a path to a non-goal position (the blue island in (c)).

A more subtle way to assure a high planning performance, beside increasing the readout time, is to increase the oscillation frequency of the planning neurons by injecting stronger external input currents. Fig 5a shows a comparison of the performance versus noise curve for a slow and a fast periodic traveling wave, and for a solitary wave. The performance is best for the fast traveling wave at a given noise level. Two reasons contribute to this effect. First, a high oscillation frequency requires stronger external input currents, and the neuron is shifted from the noise-driven regime into the drift-dominated regime where spike timing becomes more precise [20]. Second, the steady state with its phase-locking pattern represent an attractor of the phase dynamics and this attractor becomes more stable with higher frequency and hence cleans up the noise [21].

When the frequency goes to zero, the periodic wave degenerates to a single solitary wave which propagates once through the network. For this solitary wave, both benefits of a fast periodic wave disappear. First, because the spiking must be triggered by a few neighboring neurons, the planning neurons need to be in a subthreshold but depolarized regime, where they are also sensitive to noise. Second, the timing pattern of a single wave cannot profit from the phase attractor property of a periodic wave. Correspondingly, the directional information in the spike timing order for a single solitary wave quickly degrades with increasing noise level (lowest curve in Fig 5a).

To further compare the directional information in a periodic traveling wave and a solitary wave we were considering the spike timing map relative to the goal neuron for these two cases. As expected, the spike timings for the solitary wave does not fully reflect the distances to the goal, as e.g. read off from the isolated blue island in panel c.

### Planning time increases with distance and frequency

**Fig 6:**
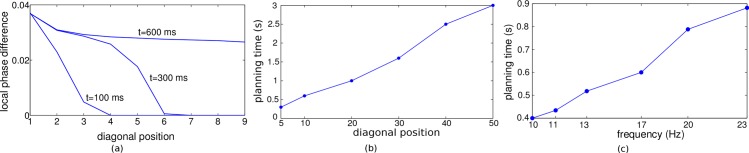
Determinants of the planning time. (a) Snapshots of the local phase differences along diagonal positions from the goal. The intrinsic oscillation frequency was 17 Hz for the non-goal positions and 18 Hz for the goal position. Noise level was *σ*_*ext*_ = 0. (b) Planning time (i.e. the time to reach roughly 90% of the final local phase difference) increases linearly with the distance from the goal. (c) Planning time also increases with the frequency of the intrinsic oscillation.

The characteristic feature of encoding the directional information in local phase differences is that, in the steady state, this information does not decay throughout the whole network (Figs 1b and 2c). However, the time to reach a steady state in the local phase differences (i.e. the planning time) increases with the distance from the goal, measured along the shortest path. While, at positions close to the goal, local phase differences are already at steady state, further away, neighboring neurons are still firing synchronously (Fig 6a). Fig 6b shows the time needed for the full phase difference to spread across a 2-dimensional network without obstacles. In the case of obstacles, the planning time increases linearly with the length of the shortest path to the goal.

Importantly, planning time also increases with the oscillation frequency (Fig 6c). This is because for increasing frequencies the excitatory postsynaptic potentials start to fall into the refractory period of the previous postsynaptic spike, and they are therefore less efficient in advancing the phase of the postsynaptic neuron [22, 23]. Ultimately, disproportionally more cycles are needed to reach steady state. One might think of increasing the synaptic strength at higher frequencies to speed up the convergence. Then the steady state is indeed reached earlier, but the stronger coupling (*ϵ*) of the neurons reduces the final phase differences [17]. Hence, in the presence of noise, planning performance again decreases (data not shown). Both, the coupling strength and the oscillation frequency, can be chosen to optimize the trade-off between better planning performance (Fig 5a) and longer planning time (Fig 6c). As we have optimized this trade-off, the relation between planning time and network size (measured along the diagonal, Fig 6b) yields a prediction of network size involved in behaviorally estimated planning times.

### Planning in a complex and changing environment

**Fig 7:**
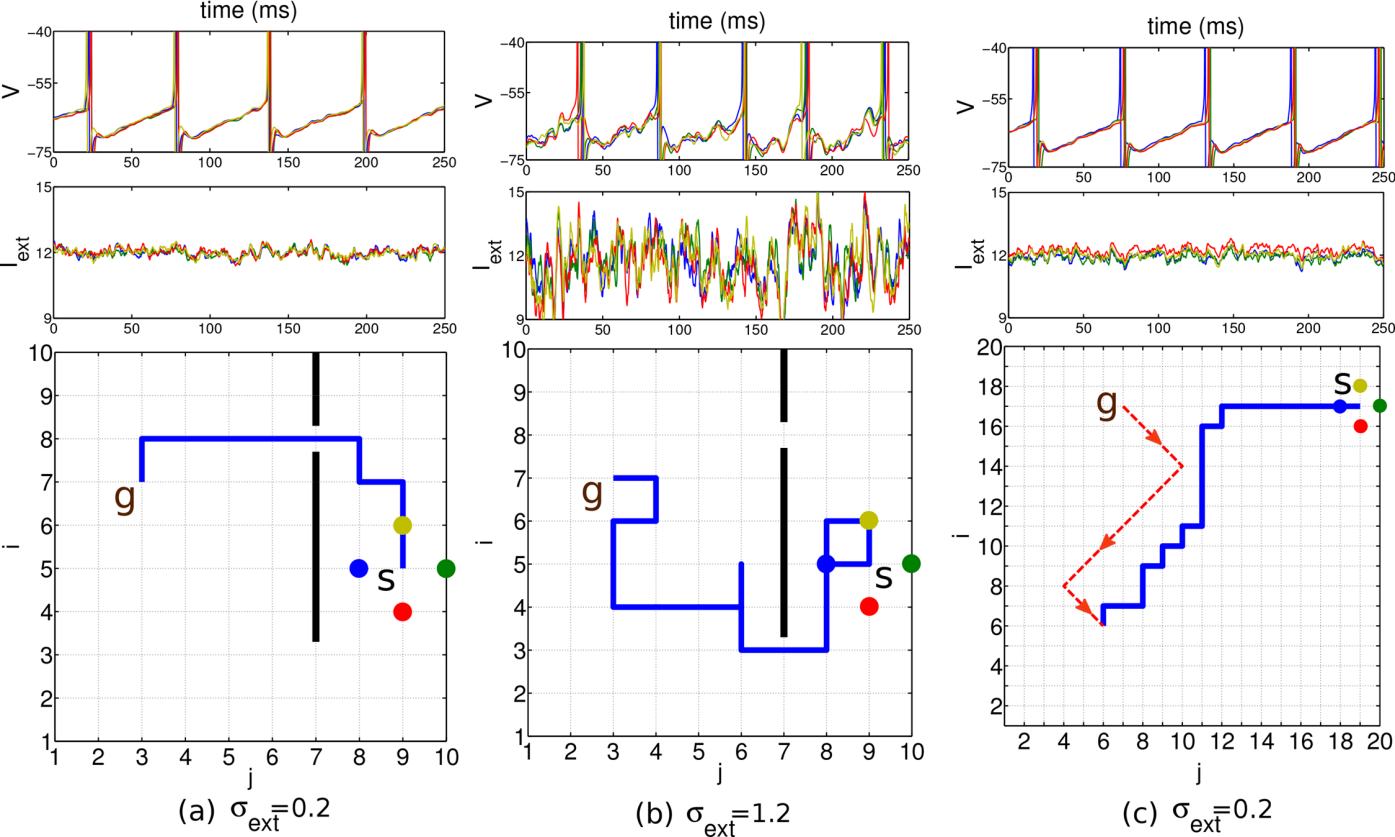
Finding shortcut and adaptive planning. (a, b) Turning up the noise level in the external input (indicated below each column) may be a way to prevent the detection of a shortest path. Other parameters as indicated in the caption of Fig 3. (c) Adaptive planning for a moving goal. A goal at initial goal position (17,7) is moved along the red line after planning time is over and the agent heads from the start towards this goal position. The goal moves one step at each readout cycle. The moving target can reliably be traced (blue line). The planning time is 1 s and the readout time is 250 ms. $\mu_{ext}^{goal}=12.5$, *μ*_*ext*_ = 12.

**Fig 8:**
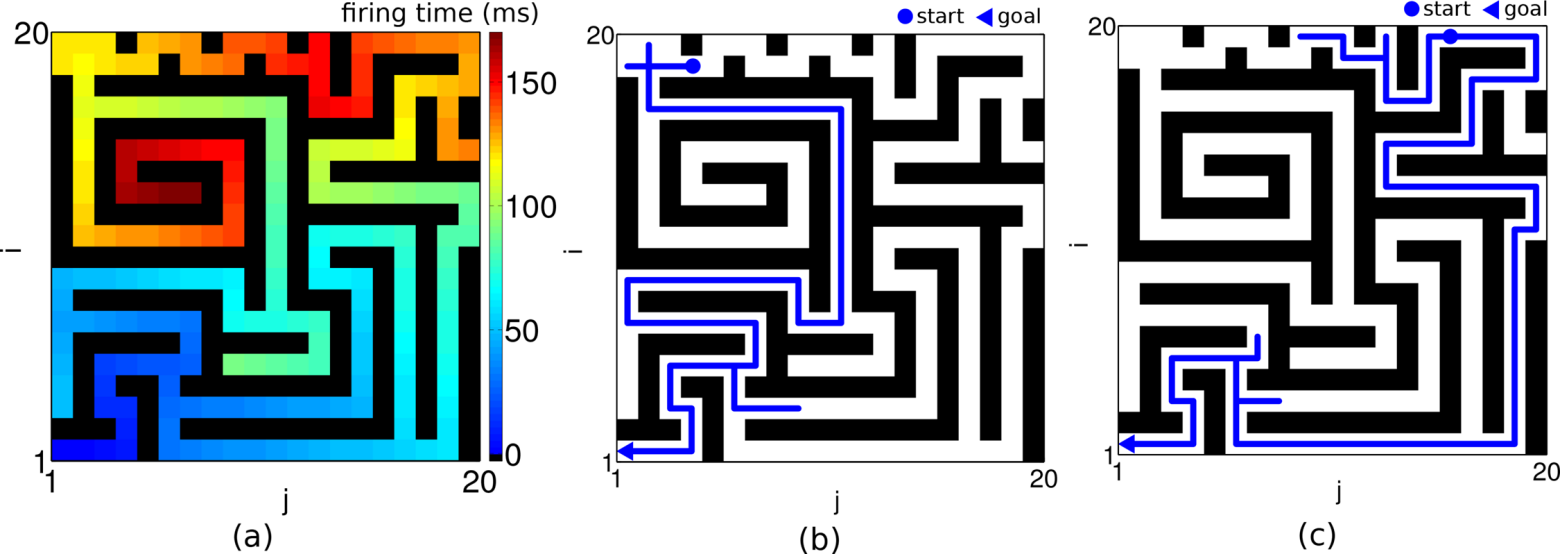
Planning in complex environments. (a) Propagated wave of activity from the goal at (1,1) through a 20 × 20 network with obstacles (black bars) is demonstrated in space-space color coded plot of firing times relative to the goal neuron. The intrinsic frequency of the goal neuron is 18 Hz. (b, c) Two examples of navigation path (blue lines) in the network from different start positions (•) to the same goal (◂) such that a slight shift of the starting position implies an entirely different shortest path. The input to the planning layer is $\mu_{ext}^{goal}$ = 12.5 and *μ*_*ext*_ = 12 and the noise level is *σ*_*ext*_ = 0.7. The planning time takes 1 s and readout time is 150 ms.

To test our network with more challenging problems we considered classical path finding tasks that have also been suggested to rate animal intelligence [24]. Whether our agent finds the shortest path through a narrow hole in an obstacle depends on the size of the hole, and on the level of noise present in the planning network. While for small noise the slippage can be found without problems (Fig 7a), increasing the fluctuations in the external synaptic input to the planning neurons precludes the finding of the shortcut (Fig 7a, 7b). We next wondered whether the network can deal with a moving goal that changes its position while the agent is on its way. This is in fact possible without pausing to wait until the network relaxes to the new steady state. Once in the steady state, a continuous displacement of the goal leads to a continuous adaptation of the firing phases of the individual planning neurons, and a direct path to the moving target is found on the fly without delay (Fig 7c). Such faithful online modifications of the optimal path would be difficult to explain if the direction field were represented by asymmetric connections that are subject to synaptic plasticity obeying its own dynamics (e.g. by anti-STDP, see [12]). Finally, we challenged the network by a complex maze where a slight shift of the starting position implies an entirely different shortest path (Fig 8). Planning times, readout times and performance were as in an open environment.

## Discussion

We have shown how directional information towards a goal can spread without information loss in a topographical network when the information is encoded in local phase differences of periodically firing neurons. In response to an additional drive of a selected goal neuron the network activity robustly self-organizes with a positive non-zero local phase difference even at large distances from the goal neuron. At any position in the network the direction of the shortest path to the goal is towards the neighbor with the smallest firing phase. The lower bound of these phase differences across the network guarantees a scale-free planning such that in the steady state, even arbitrarily far from the goal, a minimal directional information can be read out (S1 Text).

The backward spread of directional information from a goal location [7–12, 25] or both the backward spread from the goal and forward spread from the start [6] have earlier been suggested as strategies for navigational planning. However, these previous models suffer from the problem of a strong spatial information decay that causes them to break down in the presence of realistic neuronal noise. Based on the suggested phase coding, a scale-free and reliable planning now becomes possible within a single network. We have shown that the attractor property of the phase locking state makes the coding scheme robust against background noise generated from the stochastical firing of external neurons. Higher noise levels can be countered by additional readout cycles to be evaluated per action decision.

Spatial planning has been suggested to involve neurons in the medial prefrontal cortex (mPFC) that could be responsible for goal encoding [26]. In fact, impaired planning performance after mPFC lesioning [27], and human activity mapping during detour tasks, confirm the engagement of this area in navigating towards a goal [28, 29]. Wherever the goal encoding neurons reside, our theory predicts that they should display a well-timed firing pattern as part of the direction encoding network. Crucially, this network does not need to uniformly tile the whole free space of an environment. Instead, the nodes of the planning network typically represent critical spots where directional decisions need to be taken, as in the vicinity of an obstacle or at crossing or bifurcation points of paths. If the synaptic strengths within the planning network are constant, the algorithm will find the shortest path within the graph of these nodes. While the shortest graph-theoretic path may not match with the shortest Euclidean path in the 2-dimensional environment, varying the synaptic strengths may convey additional distance information. For instance, a stronger synaptic strength will advance the firing of the postsynaptic neuron, and by virtue of moving towards earlier spikes, this implicitly tells that the path across this postsynaptic neuron is shorter.

Although we are considering a planning network with nearest neighbor connectivity, this network may still represent only a sparse selection of a larger network with many more neurons around each of our planning cells. When changing the environment, another sub-network with neurons that are physically close but have still only sparse overlap may be used for planning in this new environment. In a similar way as place cells in the hippocampus are remapped in a novel environment [30], the same planning neurons may be used in different spatial contexts.

Phase-coding of directional information has several hallmarks. First, the model predicts that the waiting time for an agent put into a well-explored maze until it heads off towards the home location increases with the distance, i.e. the length of the shortest path, to the home location. This waiting time corresponds to our planning time necessary for the network to settle in a steady state. Second, phase coding inherently assumes (relative) periodic firings that leads to activity oscillations, although it does not imply the existence of a global metronome (see Fig 3c and [31]). This is reminiscent to oscillatory activity during navigational planning observed in humans [32] and rats [33]. Third, to make a single decision about a new direction, oscillations must transiently build up in our planning network before the decision is taken. Such oscillations could be related to hippocampal-cortical oscillations observed when an animal must take a directional decision in front of the junction in a Y-maze [34]. Fourth, knowing the planning times of an agent that hypothetically uses our algorithm, we can predict the network size involved in this planning. If planning time is estimated from the waiting time at the start position to be 2 s, for instance, the steady state after this time is reached at roughly diagonal position 35 (Fig 6b), and this yields a squared network size of roughly 1200 neurons (or small neuronal populations), offering 1200 decision spots for the navigation in an environment. Multiple copies of such a network could provide redundancy and could therefore help to reduce the readout time, but not the planning time itself. So the limit on the network size imposed by the planning time implies a limit on the spatial resolution of the internal map.

In terms of human cognition the suggested breadth-first search algorithm may underly the pop-up effect of just ‘seeing’ the shortest path when navigating in a relatively simple environment or looking at a map thereof. In complex planning problems, however, the shortest path ceases to simply pop-up when the spatial resolution of the available network or networks becomes insufficient. We then need to resort to heuristic strategies such as defining intermediate goals to decompose the complex planning problem into a sequence of simpler tasks, such as known for the transition from parallel to sequential search and sequence representation [35, 36]. In this sense, on a behavioral level, our model predicts that planning time should at some point start to increase nonlinearly with task complexity and that this should go in hand with an increasing likelihood that the chosen path is suboptimal.

The planning model finally may be extended by a network of position cells that provides the required positional information. This would endow the model with different interesting features. Such a position network may be used for mental navigation in terms of forward replay to new or old goals. In turn, reverse replay of past sequences in such a position network could be used for learning the backward connections in the planning network that point from the goal to the start, e.g. using classical spike-timing dependent plasticity [37]. Replay activity is in fact observed in hippocampal recordings [38]. Finally, because the movement is directed towards adjacent planning cells that fire earlier and earlier in phase, a place cell that receives input from the corresponding planning neuron would similarly advance its firing phase during the movement across its place field. Yet, as far as our planning network that operates by a phase code remains hypothetical, any re-interpretation of the experimentally observed phase precession (see e.g. [1]) in terms of a directional input from a planning network must also remain speculative.

## Methods

### Model equations and parameters

We modeled the voltage dynamics of the planning neuron at position (*i*, *j*) according to

$$\begin{array}{c} C\frac{dV_{ij}}{dt}=-I_{L}^{ij}-I_{Na}^{ij}-I_{K}^{ij}-I_{M}^{ij}+I_{ext}^{ij}\left(t\right)+\epsilon I_{syn}^{ij}\;, \end{array}$$ (2)

with leak current $I_{L}^{ij}=g_{L}(V_{ij}-E_{L})$, sodium current $I_{Na}^{ij}=g_{Na}m^{3}h\;(V_{ij}-E_{Na})$, potassium current $I_{K}^{ij}=g_{K}n^{4}(V_{ij}-E_{k})$, and outward potassium current with low threshold $I_{M}^{ij}=g_{M}q\;(V_{ij}-E_{k})$. The subscript *ij* for the gating variables *m*, *h*, *n* and *q* is ignored to lighten the notation. The conductance is *C* = 1 (unitless), and the leak conductance is *g*_*L*_ = 0.2 (in units of ms^−1^). All parameters taken from [17, 39], see also Supporting Information (S2 Text). The dynamics of the ion currents, in particularly the after-hyperpolarizing current *I*_*M*_, implies that an action potential elicited by some planning neuron cannot fire a neighboring neuron that just fired before, and this guarantees a forward spread of each single activity wave throughout the network without reverberations.

The synaptic current $I_{syn}^{ij}(t)$ is obtained as a sum across the neighboring neurons (if existing),

$$\begin{array}{c} I_{syn}^{ij}\left(t\right)=g_{syn}\sum_{k=i\pm 1,\;l=j\pm 1}s_{kl}\left(t\right)\left(E_{e}-V_{ij}\right)\;. \end{array}$$

The conductance is set to *g*_*syn*_ = 1 if the place (*i*, *j*) can be reached and = 0 if it represents a fixed obstacle. The reversal potential is *E*_*e*_ = 0. The synaptic gating variable *s*_*kl*_ of the presynaptic neuron (*k*, *l*) describes the release probability as a function of the presynaptic potential, see [40] and Supporting Information (S2 Text). The external current $I_{ext}^{ij}(t)$ in Eq 2 that drives the planning neurons (*i*, *j*) is produced by *N* ^*ij*^ afferents that are stochastically selected with connection probability *c* = 0.8 from a pool of *N*_E_ external neurons, each stochastically firing with a Poisson rate of *ν* Hz. Denoting the spike times of the *n*’th afferent to neuron *ij* by $t_{n}^{sp}$, the synaptic strength by *J* and the synaptic time constant by *τ*_*s*_ (= 2 ms), the external current follows the dynamics

$$\begin{array}{c} \tau_{s}\frac{dI_{ext}^{ij}}{dt}=-I_{ext}^{ij}+J\tau_{s}\sum_{n}^{N^{ij}}\sum_{t_{n}^{sp}}\delta \left(t-t_{n}^{sp}\right)\;. \end{array}$$

This stochastic current can be characterized by its mean and standard deviation of the form $\mu_{ext}^{ij}=J\nu \tau_{s}c\;N_{\text{E}}$ and $\sigma_{ext}=J\sqrt{\frac{1}{2}\;\nu \tau_{s}cN_{\text{E}}}$, respectively [41], both in units of mV/ms. A value of $\mu_{ext}^{ij}=12.5$ and *σ*_*ext*_ = 0.7 corresponds to an average of *N*_E_ *cν* = 73′500 input spikes per seconds with a synaptic strength of *J* = 0.081 (in units of mV/ms), and for *σ*_*ext*_ = 1.5 we had *N*_E_ *cν* = 16′000 and *J* = 0.375. The case *σ*_*ext*_ = 0 was simulated by a DC current for $I_{ext}^{ij}$ with mean $\mu_{ext}^{ij}$ (Figs 1 and 2).

For each neuron encoding a position in the environment, 4 action neurons are assigned in the action layer (Fig 2a). These action neurons are described by the leaky integrate-and-fire model with dynamics of the membrane potential

$$\begin{array}{c} \frac{dV_{a_{ij}}}{dt}=-\frac{V_{a_{ij}}}{\tau_{m}}+I_{ext}^{a_{ij}}+I_{PR}^{a_{ij}}\left(t\right)+I_{RR}^{a_{ij}}\left(t\right)\;. \end{array}$$ (3)

The index *a* stands for the cardinal directions *W*, *E*, *N*, and *S* that are represented by an action neuron at each position (*i*, *j*). Moreover, *τ*_*m*_ = 20 ms is the membrane time constant, $I_{ext}^{a_{ij}}$ is a constant external input and $I_{PR}^{a_{ij}}$ and $I_{RR}^{a_{ij}}$ are synaptic inputs to action neurons *a*_*ij*_ from the planning (*P*) and readout (*R*) layer, respectively. An action neuron emits a spike whenever its potential reaches a threshold potential *V*_*thr*_ = −50 and is then instantaneously reset to *V*_*reset*_ = −65. The external input (e.g. from hippocampal place cells) to the action neurons is set to $I_{ext}^{a_{ij}}=10$ if the agent is at position (*i*, *j*) and 0 else.

The action neuron *a*_*ij*_ receives synaptic input $I_{PR}^{a_{ij}}$ from the planning layer if its corresponding neuron emits a spike (see Fig 2a). More precisely, $I_{PR}^{a_{ij}}(t)=g_{e}\;s_{\alpha (i,j,a)}(t)$ with index function *α*(*i*, *j*, *W*) = (*i*, *j* − 1), *α*(*i*, *j*, *E*) = (*i*, *j*+1), *α*(*i*, *j*, *N*) = (*i* − 1, *j*), *α*(*i*, *j*, *S*) = (*i*+1, *j*). Here, *g*_*e*_ = 0.8 is the excitatory synaptic conductance and *s*_*kl*_(*t*) is again the synaptic gating variable driven by the membrane potential of the presynaptic neuron *kl*. To insure that at most the first of the four action neurons allocated to the same position is spiking we consider the mutual inhibition among these neurons of the form

$$\begin{array}{c} I_{RR}^{a_{ij}}=g_{inh}\sum_{t_{a_{ij}}<t}\text{PSP}\left(t-t_{a_{ij}}\right)\;, \end{array}$$

with postsynaptic potential PSP(*t*) = (*t*/*τ* ^2^)exp(−*t*/*τ*)Θ(*t*) characterized by *τ* = 2 ms, *g*_*inh*_ = −20 and step function Θ(*t*) = 1 for *t* > 0 and Θ(*t*) = 0 else. The sum is taken over all spikes emitted by presynaptic neuron *a*_*ij*_ at times *t*_*a*_*ij*__.

### Scale-free planning: Non-decaying local phase differences

Here we give an intuitive account of why the local phase difference in the steady state is strictly positive throughout a network of arbitrary size. Let us assume that the local phase differences at some position were zero, i.e. the oscillator fire synchronously with its neighbors. In this case no interaction is possible and the oscillators fire with their common intrinsic frequency. But this contradicts the fact that the intrinsic frequency of the goal is higher than others, and that in the steady-state of a periodic traveling wave all oscillators fire with the same frequency. Next, to reach a common oscillation frequency, the difference between the driving currents of the goal and the remaining neurons, $I_{ext}^{goal}>I_{ext}$, needs to be compensated by different firing phases. As the individual synaptic currents have a strictly positive initial slope, we conclude that the firing phase difference between the current position and its neighbors can not be smaller than a fixed positive value. For an analytical explanation see Supporting Information (S1 Text).

## Supporting Information

S1 Text Non-fading directional information in the planning network: Mathematical proofs. (PDF)

S2 Text Details on the HH-type model. (PDF)
