# Supplementary material for: Scale-Free Navigational Planning by Neuronal Traveling Waves
Source: PLoS One. 2015 Jul 9;10(7):e0127269. doi: 10.1371/journal.pone.0127269 (PMC4497724; doi:10.1371/journal.pone.0127269)
Supplement: S1 Text — (PDF) [file pone.0127269.s001.pdf]

S1 Text

Azadeh Khajeh-Alijani, Robert Urbanczik, Walter Senn

Non-fading directional information in the planning network: mathematical proofs

Here we study the existence of periodic traveling waves in networks of coupled neural oscillators that spread out from a goal location. We prove that at any position of the network, there is a minimal, strictly positive local phase difference that points the direction towards the goal, independently of the network size.

Traveling waves in neuronal networks

The dynamics of coupled nonlinear neuronal oscillators using conductance-based neurons such as of the Hodgkin-Huxley (HH) type have extensively been studied [1–6]. A basic question concerning the dynamics of these coupled oscillators is when phase locking can occur, i.e. when a stable periodic solution exists for which all the neuronal oscillators generate action potentials with a common period. The dynamics of such systems typically can not be solved analytically. However, in the case of weak coupling, averaging theory [7] can be used to reduce the system to a phase model for which the analysis is much simpler [3, 5, 8]. For the reduced phase model, the relevant dynamical variables are the local phase differences between the oscillators, and the effective interaction between oscillators only depends on these. Yet, the calculation of the interaction function, the so-called phase resetting curve, typically needs to be numerically evaluated [1, 3, 6].

Another class of studies considers synaptically coupled chains of Integrate-and-Fire (IF) oscillators [9–12]. These work specify conditions for the existence of stable phase-locked

Email address: azadeh.alijani@gmail.com (Azadeh Khajeh-Alijani, Robert Urbanczik, Walter Senn).

solutions that are valid for arbitrary coupling strengths. IF neurons may be regarded as a reduction of HH-type neurons, capable for generating realistic spike trains [12,13]. The discontinuous nature of the IF model allows for a rich dynamical behavior and at the same time simplifies the analysis of the dynamics and the characterization of phase locking solutions. In fact, conditions for the convergence of the dynamics of coupled IF oscillators to a steady state solution can explicitly be formulated [14, 15]. Therefore, instead of studying traveling waves with HH-type neurons (see Eq.1 in the main text), we shall consider coupled IF neurons. We follow [16] and [12] in analyzing traveling waves in 1D chains and 2D arrays of synaptically coupled IF neurons in the oscillatory regime.

Starting with a synchronous state of the coupled IF oscillators we consider a step-like current injection into the IF neuron that represents the goal location. This perturbation speeds up the goal oscillator and initiates waves of action potentials traveling throughout the network. The activity eventually converges a periodic traveling wave, i.e. to a phase-locked solution where again all frequencies are the same, but with firing phases that increase with the distance from the goal. We show that in this steady state, local phase differences between neighboring oscillators have a strictly positive lower bound at any position of the network. In the context of navigational planning, this implies that in the whole network there is always a minimal amount of directional information to find the shortest path towards a goal.

One-dimensional chains of IF neurons

We consider a chain of N synaptically coupled IF oscillators. The subthreshold dynamics of each oscillator is governed by

$$\frac{\mathrm{d}V_i}{\mathrm{d}t} = -\frac{V_i}{\tau_m} + I_i + \epsilon I^i_{syn}(t), \quad i = 1 \cdots N\,, \tag{1}$$

where V_i is the membrane potential of the i-th neuron, τ_m denotes the membrane time constant, I_i is the external input to neuron i, $I^i_{syn}(t)$ is the total synaptic current from nearest neighbors of neuron i, and $\epsilon \geq 0$ is the coupling strength. For convenience, we set $\tau_m = 1$. Each neuron fires a spike whenever its voltage reaches the threshold potential $V_{th} = 1$; thereafter it is instantaneously reset to $V_{re} = 0$ for a time period T_{ref} (absolute refractory period). We assume $I_i > 1$ so that in the absence of coupling ($\epsilon = 0$) each oscillator fires at a rate $1/T_i$, with $T_i = T_{ref} + \ln(I_i/(I_i - 1))$. The synaptic input to neuron i from its nearest-neighbor neurons j is

$$I^i_{syn}(t) = \sum_{j=i\pm1} \sum_n \alpha(t - t^n_j), \tag{2}$$

where the second summation runs over all the spikes emitted prior to time t by presynaptic neuron j at times t_j^n and $\alpha(t) = (t/\tau^2)\exp(-t/\tau)\Theta(t)$, with τ the decay time constant and with $\Theta(t) = 1$ for $t \geq 1$ and $\Theta(t) = 0$ for $t > 0$. To ensure that on a time interval of a few milliseconds the activity spreads uni-directionally between neighboring neurons we assume that the neurons only consider the synaptic inputs arriving within their non-refractory period, and hence that the refractory time is absolute. Note that this assumption approximates the effect of mainly the after-hyperpolarizing potassium current I_M in the HH-type model presented in the main text.

We want to find conditions for the existence of traveling waves. A (periodic) traveling wave is a phase-locked state in which all the neurons fire at regular intervals of length T but firing times are shifted according to a phase. Following [12], we define a phase-locked solution to be a self-consistence solution to Eqs. (1, 2) in which the n-th firing times of neuron i satisfies $t_i^n = (n+\theta_i)T$, where T is the collective period of the network and $0 \leq \theta_i < 1$ is the phase of neuron i. Under such ansatz for the firing times, we integrate Eq. 1 between two successive firing times $\theta_i T$ and $T + \theta_i T$ and obtain

$$\frac{1}{1-\mathrm{e}^{-T+T_{ref}}} = I_i + \frac{\epsilon}{\mathrm{e}^{T} - \mathrm{e}^{T_{ref}}} \int_{T_{ref}}^{T} \mathrm{e}^{s} I_{syn}^{i}(s+\theta_i T)\,\mathrm{d}s\,. \tag{3}$$

Now, using Eq. 2 and the assumption of ignoring input during the absolute refractory time, we obtain

$$\begin{aligned} I_{syn}^{i}(s) = &\sum_{j=i\pm 1} \alpha(s - T - \theta_j T)\,\cdot \\ &\cdot \Big[\Theta(T - T_{ref} + (\theta_j - \theta_i)T) - \Theta((\theta_j - \theta_i)T)\Big]\,, \end{aligned} \tag{4}$$

with the step-function Θ defined above. Eq. 3 then becomes

$$\frac{1}{1-\mathrm{e}^{-T+T_{ref}}} = I_i + \sum_{j=i\pm 1} K_T(\theta_i - \theta_j), \tag{5}$$

where

$$\begin{aligned} K_T(\phi) = &\,\epsilon(\mathrm{e}^{T} - \mathrm{e}^{T_{ref}})^{-1}\,\cdot \\ &\cdot \int_{T_{ref}}^{T} \mathrm{e}^{s}\alpha(s - T + \phi T)\Big[\Theta(T - T_{ref} - \phi T) - \Theta(-\phi T)\Big]\,\mathrm{d}s\,. \end{aligned} \tag{6}$$

We next abbreviate $\Omega = 1/(1-\mathrm{e}^{-T+T_{ref}})$ and the local phase difference $\phi_i = \theta_{i+1} - \theta_i$. The conditions for the phase locking solutions of a chain of IF oscillators then read

$$\begin{aligned} &\Omega = I_1 + K_T^{+}(-\phi_1), \\ &\Omega = I_i + K_T^{+}(-\phi_i) + K_T^{-}(\phi_{i-1}), \quad i = 2\cdots N-1 \\ &\Omega = I_N + K_T^{-}(\phi_{N-1})\,, \end{aligned} \tag{7}$$

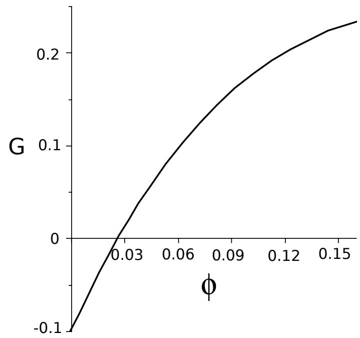

Figure 1. *Plot of $G(\phi) = I - I_1 + K_T^-(\phi)$, Eq. 10, as a function of the local phase difference ϕ. A stable solution corresponds to zero crossing of G with a positive slope. The graph in particularly shows that the synaptic interaction function $K_T^-(\phi)$ ($\equiv K_T(\phi)$, Eq. 6) is strictly increasing for small ϕ, with $K_T^-(0) = 0$. Parameter values: $I_1 = 1.4$, $I = 1.3$, $\tau = 2\tau_m$, $\epsilon = 0.5$, $T_{ref} = 5\tau_m$, and $T = 6.25\tau_m$.*

with boundary conditions $K_T^-(\phi_0) \equiv 0 \equiv K_T^+(-\phi_N)$. Here $K_T^- = K_T^+ = K_T$, and the superscripts $(-)$ and $(+)$ are merely referring to two summands $j = i-1$ and $j = i+1$ in Eq. 5 and thus indicate whether the interactions from the left or right neighbor of neuron i, respectively, is considered.

Of particular interest from the perspective of planning is a traveling wave solution in which the firing phase increases monotonically from the goal neuron along the chain. For the rest of this Section we shall assume that the neuron at position 1 represents the goal neuron. It receives the input $I_1 > I > 0$, while all other neurons receive the identical input ($I_i \equiv I$, $i = 2 \cdots N$). Solutions to Eq. 7 define the local phase difference of oscillators, ϕ_i , and the collective frequency of oscillations, $1/T$. These solutions a priori include both synchronous and traveling waves. But a synchronous state can be excluded as substituting $\phi_i = 0$ ($i = 1 \cdots N$) in the above system, and using the fact that $K_T^+(0) = K_T^-(0) = 0$, results in $I_1 = I$ which contradicts our assumption.

We next show that the remaining traveling wave solution has a fixed local phase difference across the network. Due to the additional drive of the goal neuron at position 1, and because the absolute refractory time covers the time difference between the firing of neighboring neurons, there is no backward interaction, i.e. neuron i is only affected by its left neighbor $j = i - 1$. This implies that $K_T^+(\dots) = 0$ in the system (7). Next, because the synaptic drive $\alpha(t)$ is a monotonically strictly increasing function for small $t > 0$, the same holds for $K_T^-(\phi)$ for small $\phi > 0$, see Fig. 1. Because $I_i \equiv I$ for all $i \geq 2$ each of the equations for $i \geq 2$ is therefore solved by a unique local phase difference

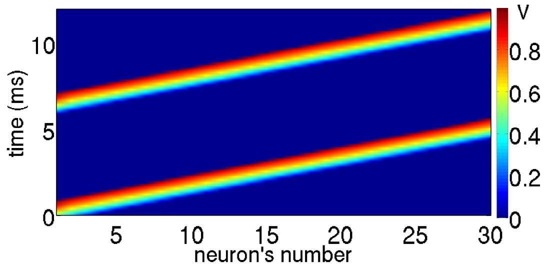

Figure 2. *Traveling wave in a chain of 30 IF neurons induced by a step current injection in neuron 1 (Eq. 1). Colors encode voltage at steady state. The collective period and the local phase differences are* $T = 6.25\tau_m$ *and* $\phi = 0.026$*, respectively. These numbers correspond to the solutions obtained from Eqs 8 and 9.*

$\phi_{i-1} = \phi > 0$. Hence, the system (7) of N equations is reduced to the two equations,

$$\Omega = I_1 \tag{8}$$
$$\Omega = I + K_T^-(\phi)\,, \tag{9}$$

where $\phi \equiv \phi_{i-1}$ for $i = 2 \cdots N$. Note that these two equations are equivalent to the phase-locking conditions for a pair of IF oscillators. From Eq. 8, using the definition of Ω, the population frequency $1/T$ is found, and with Eq. 9 this implies that all the neurons in the steady state oscillate with the same frequency as that of the goal neuron. Moreover, Eq. 9 gives the phase-locking solution ϕ, indicating that at steady state there is a constant firing phase difference between neighboring neurons.

To determine stability of the solution we subtract (8) from (9) which gives the condition

$$G(\phi) = I - I_1 + K_T^-(\phi) = 0\,. \tag{10}$$

Figure 1 shows a plot of $G(\phi)$; as can be seen there is only one solution of Eq. 10, $\phi = 0.026$. This solution corresponds to a traveling wave in which firing phases are monotonically increasing from neuron 1 along the chain with fixed local phase difference ϕ (see Fig. 2). For a pair of IF oscillators, [16] obtained a condition for the stability of a phase-locking solution as $\partial_\phi G(\phi) > 0$. This condition holds here too, implying the stability of the phase-locking solution ϕ for the chain of IF oscillators. The speed of the wave propagation as discussed in [17] depends on various aspects of the model and on the dynamics and strength of the synapses. Here, it depends on ϵ, τ and $I_1 - I$, but importantly, it is independent of the chain length (the solutions ϕ and T are valid for any N).

Figure 2 shows a traveling wave in a simulation with 30 IF neurons in a chain, induced by a frequency difference between goal neuron 1 ($I_1 = 1.4$) and the other neurons ($I =$

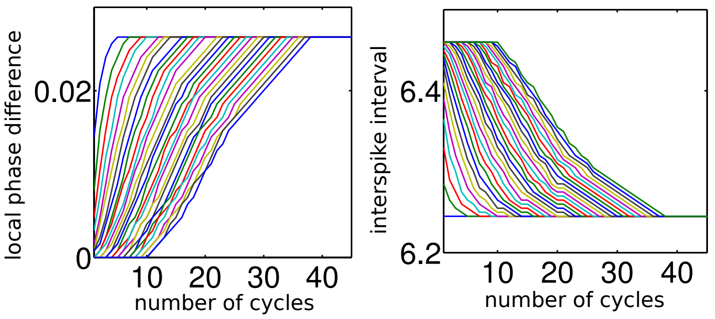

Figure 3. *Evolution of the local phase differences (a) and interspike intervals (b) for neurons 1 to 30 (represented by the 30 colored lines appearing from left to right) as a function of the number of collective cycles of length T. Both local phase differences and interspike intervals reach the steady state after 40 cycles. Note that the local phase difference of a fixed neuron i from its neighbor $j = i - 1$ monotonically increases in time, i.e. with each cycle of the oscillation (as represented by a single curve in (a)).*

1.3). The color coding reflects membrane potential of the neurons after the transient period. The frequency difference is propagated from neuron 1 throughout the chain with a constant increase in firing time, $\phi\, T = t_{i+1}^n - t_i^n = 0.165$. In Figure 3a we show the dynamics of the local phase differences ϕ_i of neurons 1 to 30 (corresponding to the 30 lines from left to right, respectively) as a function of the number of collective cycles. All the neurons reach the steady state value of the phase difference at $\phi = 0.026$, as it was predicted by the solution of Eq. 10. In Figure 3b the interspike intervals of each oscillator (represented again by lines) is plotted as a function of the number of collective cycles. In the steady state, all neurons reached the population period $T = 6.25\tau_m$ after nearly 40 periods of firing. The same value of T is obtained by solving Eq. 8 for the parameters given in the caption of Fig. 1.

Two-dimensional arrays of IF neurons

We next consider a $N \times N$ array of coupled IF oscillators in which each oscillator is connected with its four neighbors. We define the local phase differences of neuron ij to the east and north neighbor, respectively, by

$$\begin{aligned} \psi_{ij} &= \theta_{i,j+1} - \theta_{ij}, \quad i = 1 \cdots N, \quad j = 1 \cdots N-1 \\ \phi_{ij} &= \theta_{i+1,j} - \theta_{ij}, \quad i = 1 \cdots N-1, \quad j = 1 \cdots N \end{aligned} \tag{11}$$

Following the corresponding arguments from the 1D case presented above, we obtain the phase-locking conditions for the 2D-array of coupled IF oscillators in the steady

state,

$$\Omega = I_{ij} + K_T^{x+}(-\psi_{ij}) + K_T^{x-}(\psi_{i,j-1}) + K_T^{y+}(-\phi_{ij}) + K_T^{y-}(\phi_{i-1,j}), \tag{12}$$

$(i, j = 1 \cdots N)$ with the K_T's representing the interaction functions defined in Eq. 6, and the superscripts $x+$ and $x-$ ($y+$ and $y-$) indicating whether the synaptic drive of neuron ij is coming from the east and west (north and south) neighbor, respectively. Here, the cardinal directions refer to the 2D plot of the network (e.g. in Fig. 2b and 3 of the main text) with the goal neuron (1,1) displayed in the bottom-left (= south-west) corner. I_{ij} is a fixed external input to the neuron at position (i, j). For the boundary neurons Eq. (12) is subject to the conditions $K_T^{y-}(\phi_{0j}) \equiv K_T^{x-}(\psi_{i0}) \equiv K_T^{y+}(-\phi_{N,j}) \equiv K_T^{x+}(-\psi_{i,N}) \equiv 0$.

As in the 1D case, we consider an input current $I_{ij} = I$ to all IF neurons except the goal neuron, for which the input current is $I_{11} > I$. The fact that the external drive of the goal neuron is stronger than that for the other neurons, together with the assumption of an absolute refractory period, implies that the effective interactions are directed from the goal neuron to the subsequent east and north neighbors (i.e. in the direction of higher indices). The system of equations (12) therefore becomes

$$\Omega = I_{11} \tag{13}$$
$$\Omega = I + K_T^{x-}(\psi_{1j}), \quad j = 1 \cdots N-1 \tag{14}$$
$$\Omega = I + K_T^{y-}(\phi_{i1}), \quad i = 1 \cdots N-1 \tag{15}$$
$$\Omega = I + K_T^{x-}(\psi_{i,j-1}) + K_T^{y-}(\phi_{i-1,j}), \quad i, j = 2 \cdots N. \tag{16}$$

Note that the symmetry of the interactions in the south-to-north and west-to-east direction ($K_T^{y-} = K_T^{x-}$) results in a symmetry of phase differences along these directions, $\phi_{ij} = \psi_{ji}$, respectively. Eqs 14 and 15 corresponding to the first row and first column of neurons, respectively, are therefore reduced to the 1D case (Eqs 7 and 9, respectively),

$$\Omega = I + K_T^{-}(\phi_{i1}), \tag{17}$$

with $\phi_{i1} = \psi_{1i}$ for $i = 1 \cdots (N-1)$, and $K_T^{y-} = K_T^{x-} = K_T^{-} = K_T$ as defined in Eq. 6. Since K_T^{-} is monotonically increasing, there is again a unique solution $\phi_{i1} = \phi_1 > 0$ for Eq. 17, and this holds for any network size N.

For neurons positioned off from the network boundary, the south-to-north and west-to-east interactions add up and the total input current in Eq. 16 reduces to

$$\Omega = I + K_T^{-}(\psi_{i,j-1}) + K_T^{-}(\phi_{i-1,j}).$$

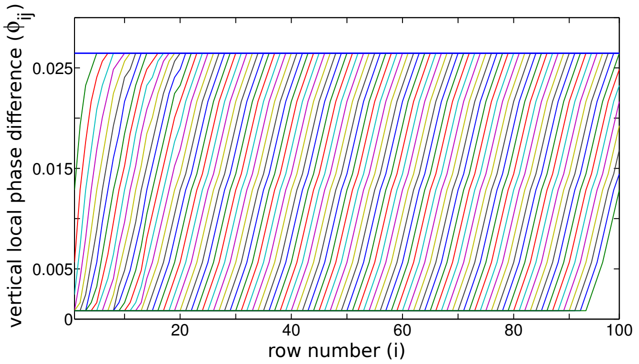

Figure 4. *Plot of the vertical local phase differences ϕ_{ij} in an array of 100×100 IF oscillators at steady state. Top line represents the local phase differences in the first column, $\phi_{i1} = \phi_1$. Each line from left to right represents the vertical local phase differences ϕ_{ij} for fixed column index j as a function of the row number i. The index j of the curves increases from very left ($j = 1$) to very right ($j = 100$). The steady-state local phase difference in 2D is also strictly positive, with upper bound $\phi_1 = 0.026$ and a lower bound $\phi_{100} = 8 \times 10^{-4}$.*

Since $\Omega = I_{11}$ (Eq. 14) we conclude that

$$K_T^-(\psi_{i,j-1}) + K_T^-(\phi_{i-1,j}) = I_{11} - I > 0\,, \tag{18}$$

independently of the position (i, j) of the neuron and independently of the network size N. Since $K_T^-(\phi)$ for small ϕ is strictly increasing, see Fig. 1, there is some $\gamma > 0$ (depending on the synaptic transmission and neuron parameters) such that in the relevant regime $K_T^-(\phi) \geq \gamma\,\phi$. Hence, from Eq. 18 we conclude that at any position (i, j) of the network the local phase differences to the west ($\psi_{i,j-1}$) and south ($\phi_{i-1,j}$) neighbor in the steady state are summed up to a strictly positive value,

$$\psi_{i,j-1} + \phi_{i-1,j} \geq (I_{11} - I)/\gamma > 0\,. \tag{19}$$

Further, due to the strictly positive slope of $K_T^-(\phi)$, this phase-locked solution is stable. In fact, a positive perturbation of the phase to the east or north (corresponding to a delayed firing of neuron ij with respect to the west or south neighbor) would strengthen the interaction $K_T^-(\psi_{i,j-1})$ and $K_T^-(\phi_{i-1,j})$, respectively, and speed up the firing of neuron ij, thereby correcting for its delay. Conversely, a negative perturbation from the steady-state phase (corresponding the an earlier firing of neuron ij) would imply a decrease of the interaction strength and would therefore correct the perturbation by delaying the firing of neuron ij again.

Since these local phase differences encode the direction towards the goal, we conclude from inequality (19) that in the steady state there is always a strictly positive directional information throughout the network. Remember that the four action neurons at a given position (i, j) read out which of the four neighboring neurons fires first, and then elicit

the action to move towards the position corresponding to that neuron. Hence, an animal at position (i, j) will choose to move towards position $(i, j-1)$ or $(i-1, j)$, depending on whether the local phase difference $\psi_{i,j-1}$ or $\phi_{i-1,j}$ is larger, i.e. whether neuron $(i, j-1)$ or $(i-1, j)$, respectively, fires earlier. Note also that due to the goal position at $(1, 1)$ the neurons at positions $(i, j+1)$ and $(i+1, j)$ 'behind' neuron ij fire later than neuron ij, and hence these directions are not chosen.

In Figure 4 we show a simulation of the vertical local phase differences $\phi_{i,j}$ in an array of 100×100 coupled IF neurons at steady state. Each line in the plot corresponds to $\phi_{i,j}$ for a fixed column j. The upper bound is determined by the local phase differences along the first column, $\phi_1 = \phi_{i,1} = 0.026$ (top line). The same value of the local phase difference is obtained by solving Eq. 17. The vertical local phase difference ϕ_{ij} between neurons in the next columns $j = 2 \cdots 100$, as a function of the row number i, are shown by the lines from left to right, respectively. The last column determines the lower bound $\phi_{100} = 8 \times 10^{-4}$. Hence, the results from the 1D case also carry over the the 2D case where in the steady state there is again a lower positive bound for the local phase differences.

References

[1] Ermentrout GB, Kopell N. Frequency plateaus in a chain of weakly coupled oscillators, I. SIAM J Math Anal. 1984;15:215–237.

[2] Kopell N, Ermentrout GB. Symmetry and phase-locking in chains of weakly coupled oscillators. Comm Pure Appl Math. 1986;39:623–660.

[3] Kopell N. Toward a theory of modelling central pattern generators. In: Cohen A, editors. Neural control of rhythmic movements in vertebrates. New York, Wiley. pp. 369–413; 1988.

[4] Kopell N, Ermentrout GB. Phase transitions and other phenomena in chains of coupled oscillators. SIAM J App Math. 1990;50(4):1014–1052.

[5] Ermentrout GB, Kopell N. Multiple pulse interactions and averaging in systems of coupled neural oscillators. J Math Biol. 1991;29(3):195–217.

[6] Hansel D, Mato G, Meunier C. Phase dynamics for weakly coupled Hodgkin-Huxley neurons. Europhys Lett. 1993;23(5):367–372.

[7] Guckenheimer J, Holmes P. Nonlinear oscillations, dynamical systems, and bifurcations of vector fields. Springer-Verlag; 1983.

[8] Kuramoto Y. Chemical Oscillations, Waves and Turbulence. Springer, New York; 1984.

[9] Bressloff PC, Coombes S. Traveling waves in chain of pulse-coupled oscillators. Phys Rev Lett. 1998;80(21):4815–4818.

[10] Bressloff PC, Coombes S. Desynchronization, mode-locking and bursting in strongly coupled integrate-and-fire oscillators. Phys Rev Lett. 1998;81(10):2168–2171.

[11] Bressloff PC, Coombes S. Spike train dynamics underlying pattern formation in integrate-and-fire oscillator networks. Phys Rev Lett. 1998;81(11):2384–2387.

[12] Bressloff PC, Coombes S. Travelling waves in chains of pulse-coupled integrate-and-fire oscillators with distributed delays. Physica D. 1999;130:232–254.

[13] Rauch A, La Camera G, Luscher HR, Senn W, Fusi S. Neocortical pyramidal cells respond as integrate-and-fire neurons to in vivo-like input currents. J Neurophysiol. 2003 Sep;90(3):1598–1612.

[14] Mirollo RE, Strogatz SH. Synchronization of pulse-coupled biological oscillators. SIAM J Appl Maths. 1990;50(6):1645–1662.

[15] Vreeswijk Cv. Partial synchronization in populations of pulse-coupled oscillators. Phys Rev E Stat Phys Plasmas Fluids Relat Interdiscip Topics. 1996;54(5):5522–5537.

[16] Van Vreeswijk C, Abbott LF, Ermentrout GB. When inhibition not excitation synchronizes neural firing. J Comput Neurosci. 1994;1(4):313–321.

[17] Ermentrout GB. The analysis of synaptically generated traveling waves. J Comput Neurosci. 1997;5:191–208.
