# Supplementary material for: Scale-Free Navigational Planning by Neuronal Traveling Waves
Source: PLoS One. 2015 Jul 9;10(7):e0127269. doi: 10.1371/journal.pone.0127269 (PMC4497724; doi:10.1371/journal.pone.0127269)
Supplement: S2 Text — (PDF) [file pone.0127269.s002.pdf]

S2 Text

Azadeh Khajeh-Alijani, Robert Urbanczik, Walter Senn

Details on the HH-type model

Here we give the details of the ionic currents and synaptic gating variables of the HH model. The ionic currents were implemented according to [1,2] and are reproduced here for the readers convenience. The gating variables $x = m, h, n$ and s obey the dynamics

$$\frac{dx}{dt} = a_x(V_{ij})(1-x) - b_x(V_{ij})x \ .$$

The parameters for the currents are E_L = -67 mV, E_{Na} = 50 mV, E_K = -100 mV, g_L = 0.2, $g_{Na} = 100$, $g_K = 80$, $g_M = 3$ (all conductances are mS/cm^2) and

$$\begin{aligned}
a_m(V_{ij}) &= 0.32(V_{ij}+54)/\Big[1-\exp(-(V_{ij}+54)/4)\Big],\\
b_m(V_{ij}) &= 0.28(V_{ij}+27)/\Big[-1+\exp((V_{ij}+27)/5)\Big],\\
a_h(V_{ij}) &= 0.128\exp(-(V_{ij}+50)/18),\\
b_h(V_{ij}) &= 4/\Big[1+\exp(-(V_{ij}+27)/5)\Big],\\
a_n(V_{ij}) &= 0.032(V_{ij}+52)/\Big[1-\exp(-(V_{ij}+52)/5)\Big],\\
b_n(V_{ij}) &= 0.5\exp(-(V_{ij}+57)/40)\Big],
\end{aligned}$$

Also for the M-current, we have

$$\tau_q(V_{ij})\frac{dq}{dt} = q_\infty(V_{ij}) - q,$$

Email address: azadeh.alijani@gmail.com (Azadeh Khajeh-Alijani, Robert Urbanczik, Walter Senn).

where

$$\tau_q(V_{ij}) = \bar{\tau}/\Big[3.3\exp((V_{ij}+35)/20) + \exp(-(V_{ij}+35)/20)\Big],$$
$$q_\infty = 1/\Big[1 + \exp(-(V_{ij}+35)/10)\Big]$$

and $\bar{\tau} = 400$ mS.

The synaptic gating variable determining $I_{syn}^{ij}(t)$ is given by [3]

$$\frac{\mathrm{d}s_{kl}}{\mathrm{d}t} = -s_{kl}/\tau_s + \frac{\alpha(1-s_{kl})}{1+\mathrm{e}^{-(V_{kl}-\bar{V})/\Delta V}} \tag{1}$$

with $\tau_s = 2$, $\alpha = 2$, $\bar{V} = -5$, and $\Delta V = 2$.

References

[1] Traub RD, Jefferys JG, Miles R. Analysis of the propagation of disinhibition-induced after-discharges along the guinea-pig hippocampal slice in vitro. J Physiol (Lond). 1993;p. 267–287.

[2] Ermentrout GB. The analysis of synaptically generated traveling waves. J Comput Neurosci. 1997;5:191–208.

[3] Golomb D, Amitai Y. Propagating neuronal discharges in neocortical slices: computational and experimental study. J Neurophysiol. 1997;78:1199–1211.
